# Increased expression of the Cbl family of E3 ubiquitin ligases decreases Interleukin-2 production in a rat model of peripheral neuropathy

**DOI:** 10.1186/s12871-018-0555-z

**Published:** 2018-07-18

**Authors:** Ji Seon Jeong, Ha Yeon Kim, Byung Seop Shin, Ae Ryoung Lee, Ji Hyun Yoon, Tae Soo Hahm, Ja Eun Lee

**Affiliations:** 1 Department of Anesthesiology and Pain Medicine, Samsung Medical Center, Sungkyunkwan University, School of Medicine, 81, Irwon-ro, Gangnam-gu, Seoul, 06351 South Korea; 2 Department of Anesthesiology and Pain Medicine, Ajou University Medical Center, Ajou University, School of Medicine, Seoul, South Korea; 3 Department of Anesthesiology and Pain Medicine, Cheju National University Hospital, Jeju National University, School of Medicine, Jeju, South Korea; 4 Department of Life Science, College of Natural Science, Chung-Ang University, Seoul, South Korea

**Keywords:** Cbl family, Interleukin-2, Neuropathic pain, Phospholipase Cγ1, Ubiquitin, ZAP70

## Abstract

**Background:**

Interleukin 2 (IL-2) influences the development and severity of pain due to its antinociceptive and immunomodulatory effects. Its production is influenced by the increased expression of c-Cbl (Casitas B-lineage lymphoma proto-oncogene) and Cbl-b E3 ubiquitin ligases. We evaluated the effects on IL-2-mediated changes in c-Cbl and Cbl-b expression in a rat model of chronic neuropathic pain.

**Methods:**

Peripheral neuropathy was induced in adult male Sprague-Dawley rats weighing 250–300 g by chronic spinal nerve ligation. Half of the spinal cord ipsilateral to the nerve injury was harvested at 1, 3, and 6 weeks, and the expression levels of IL-2, c-Cbl, Cbl-b, phospholipase C-γ1 (PLC-γ1), ZAP70, and protein kinase Cθ (PKCθ), as well as ubiquitin conjugation, were evaluated.

**Results:**

Total IL-2 mRNA levels were significantly decreased at 3 and 6 weeks after nerve injury compared to those in sham-operated rats. The mRNA levels of c-Cbl and Cbl-b, as well as the level of ubiquitin conjugation, were significantly increased at 3 and 6 weeks. In contrast, the levels of phosphorylated ZAP70 and PLC-γ1 were decreased at 3 and 6 weeks after spinal nerve ligation. Ubiquitination of PLC-γ1 and PKCθ was increased at 3 and 6 weeks.

**Conclusions:**

Our results suggest that ubiquitin and the E3 ubiquitin ligases c-Cbl and Cbl-b function as neuroimmune modulators in the subacute phase of neuropathic pain after nerve injury.

## Background

Neuropathic pain induced by peripheral nerve injury is characterized by stimulus-independent pain, hyperalgesia, and allodynia to mechanical or thermal stimulation [1]. To enable the development of effective treatments, the mechanism(s) underlying neuropathic pain caused by nerve injury have been the focus of numerous studies. Alteration of the immune response of the nervous system after nerve injury contributes to the complex symptoms of pain, [2] and abnormal immune responses not only in the peripheral nervous system but also in the central nervous system play important roles in the pathogenesis of neuropathic pain [1, 3, 4]. More specifically, T cells play a key role in the development and maintenance of neuropathic pain [5, 6]. Peripheral nerve injury induces recruitment of T cells into the peripheral lesion as well as the central nervous system. Costigan et al. reported that T cell infiltration into the dorsal horn begins 3 days after partial peripheral nerve injury and increased for up to 21 days in adult rats [7].

Previous studies have shown that Interleukin 2 (IL-2), which was named “T cell growth factor at the time of its discovery,” plays a crucial role in maintaining the immune system homeostasis by modulating the functional balance between T helper 17 (Th17) and regulatory T cells (Treg) [8, 9]. In addition to its antinociceptive effects, IL-2 can influence the development, maintenance, and severity of neuropathic pain symptoms by affecting the immune system homeostasis [8].

The casitas B lineage lymphoma (Cbl) proto-oncogene family of E3 ubiquitin ligases are key regulators of T cell function and influence the production of IL-2 by negatively regulating receptor tyrosine kinases (RTKs) signaling in T cells [10, 11]. Decreased IL-2 production, which is associated with the increased expression of c-Cbl and Cbl-b, is an indicator of suppression of T cell function [11, 12]. However, the mechanism by which IL-2 production is changed in chronic neuropathic pain, where functional changes of T cell occur, is yet to be elucidated.

In this study, we evaluated IL-2 mRNA levels in rats with chronic neuropathic pain induced by spinal nerve ligation, and the effects of changes in c-Cbl and Cbl-b expression in T cells on downstream factors in the RTK signaling pathway including ZAP 70 and phospholipase C-γ1 (PLC-γ1), which influence IL-2 production.

## Methods

All of the animal experiments were approved by the Institutional Animal Care and Committee of Samsung Biomedical Research Institute (IACUC No: 20150430001), which is a leading medical research center in terms of patient numbers and data collection. We followed the recommendations of the National Institutes of Health Guidelines for the Care and Use of Laboratory Animals.

### Spinal nerve ligation and assessment of mechanical allodynia

**Fig. 1:**
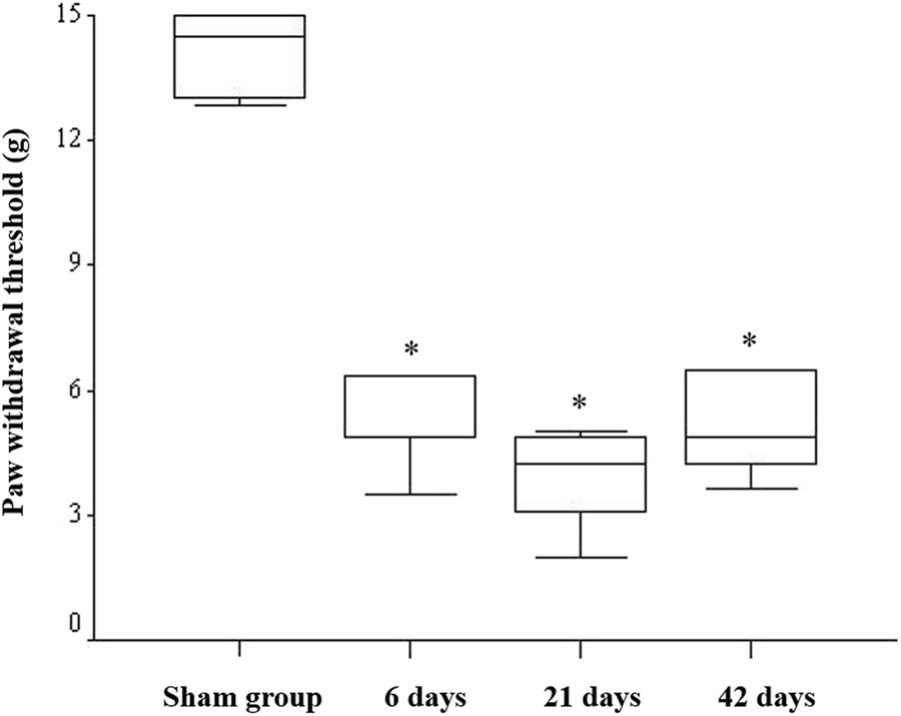
The results of von Frey mechanical allodynia test at 6, 21 and 42 days. * *P* < 0.05 versus sham-operated group

Forty-six adult male Sprague-Dawley rats weighing 250–300 g (Orient Bio, Kyunggi-Do, Korea) were used. The rats were housed in separate cages and acclimatized for at least 7 days in a controlled-temperature room before use. The room had artificial light with a 12/12 h day/night cycle, and food pellets and water were provided ad libitum. We used the rat model of chronic spinal nerve ligation-induced peripheral neuropathy described by Kim and Chung [13]. The surgical procedure was performed aseptically under inhaled isoflurane anesthesia (3% for induction and 2% for maintenance) with 100% oxygen. The lower back was shaved and a left paravertebral incision (L3–S2) was made. After resection of the left L6 transverse process, the left L5–L6 spinal nerve was ligated tightly with 6–0 black silk under a microscope. The skin wound was closed with 5–0 black silk, and bacteriostatic powder was applied topically to the wound site. The sham operation was identical, with the exception that L5–L6 spinal nerve ligation was not performed. Development of the neuropathic pain was confirmed by a withdrawal of the right hindpaw in response to a tactile stimulus using von Frey filaments (Stoelting, Wood Dale, IL, USA) at 6 days after the nerve injury and followed up at 3 and 6 weeks, as previously described [14]. Briefly, the rats were placed in a Plexiglas box with a stainless steel mesh floor and allowed to acclimatize for at least 30 min before performing the von Frey test. Eight filaments (0.41, 0.70, 1.20, 2.00, 3.63, 5.50. 8.50 and 15 g) were consecutively applied to lift the plantar surface of the right hindpaw. Withdrawal of the right hindpaw was considered a positive result, and the absence of withdrawal within 5 s was considered a negative result. Development of hyperalgesia was determined by the comparison with the baseline values, obtained from the right hindpaw during the presurgical period (*P* < 0.05) (Fig. 1). After confirming the development of neuropathic pain, the rats were randomly allocated into the 1-, 3-, or 6-week group, or the sham-operated group, and half of the spinal cord ipsilateral to the nerve injury was harvested. The 1-week timepoint was included to detect early changes in the spinal cord in acute phase because T cell infiltration begins 3 days after nerve injury. The 3-week timepoint was included as this is the time of peak T cell infiltration [7]. The 6-week timepoint represents the chronic phase of the disease.

### Western blot

Rats were deeply anesthetized by inhalation of 4% isoflurane in 100% oxygen. Next, intracardiac perfusion with cold 0.9% normal saline (4 °C) was performed, and half of the L5–L6 spinal cord ipsilateral to the nerve injury was harvested and immediately frozen in liquid nitrogen. The ipsilateral halves of the L5–L6 spinal cord were homogenized in lysis buffer (10 mM 4-(2-hydroxyethyl)-1-piperazineethanesulfonic acid [HEPES], 10 mM KCl, 0.1 mM ethylene glycol-bis(β-aminoethyl ether)-N,N,N′,N′-tetraacetic acid (EGTA), 1 mM dithiothreitol [DTT], 1 μg/mL aprotinin, 1 mM phenylmethylsulfonyl fluoride [PMSF], 10% NP-40, protease inhibitor cocktail [Sigma-Aldrich Co., St. Louis, MO, USA], and phosphatase inhibitor mixture [Phosphostop; Sigma]) and was incubated on ice for 30 min. Proteins were separated by centrifugation at 14,000 rpm for 15 min at 4 °C, and the supernatant protein concentration was quantified using protein assay reagent (Bio-Rad, Hercules, CA, USA). Equal amounts of protein (30 μg) were resolved by sodium dodecyl sulfate-polyacrylamide gel electrophoresis (SDS-PAGE) (4–15% gradient gel; Bio-Rad) and transferred onto a polyvinylidene difluoride (PVDF) membrane (Millipore, Bedford, MA, USA). The membrane was blocked with 5% skim milk in Tris-buffered saline (TBS) at room temperature for 1 h and incubated overnight (14 h) at 4 °C with the following primary antibodies: polyclonal mouse anti-Cbl-b (Santa Cruz Biotechnology, CA, USA), monoclonal mouse anti-c-Cbl (BD Biosciences, NJ, USA), monoclonal mouse anti-c-Cbl (pY700; BD Biosciences), polyclonal rabbit anti-ZAP70 (Santa Cruz), polyclonal rabbit anti-ZAP70 (pY 318; Abcam, Cambridge, UK), polyclonal rabbit anti-phospholipase Cγ1 (Santa Cruz), polyclonal rabbit anti-phospholipase Cγ1 (pY775; Abcam), polyclonal rabbit anti-PKCθ (Santa Cruz), polyclonal rabbit anti-PKCθ (pS 676; Santa Cruz), anti-mono- and poly-ubiquitinated conjugated monoclonal (Enzo Life Sciences, Farmingdale, NY, USA), polyclonal goat anti-interleukin-2 (R&D Systems, Minneapolis, MN, USA), and anti-GAPDH (Abcam). Anti-phospho-tyrosine (anti-pTyr; Merck, Darmstadt, Germany) and protein A agarose (Thermo Fisher Scientific, Waltham, MA, USA) were used to evaluate phosphorylation of Cbl-b. The primary antibody was diluted 1:1000–1:2000 in TBS with 5% skim milk according to the manufacturer’s protocol. The membranes were washed three times with TBS with 0.5% Tween 20, and incubated with corresponding horseradish peroxidase-conjugated anti-mouse IgG (1:5000; Cell Signaling Technology), anti-rabbit IgG (1:5000, Cell Signaling Technology), or anti-goat IgG (1:3000, Cell Signaling Technology) secondary antibody at room temperature for 2 h. The membranes were washed three times with 0.5% Tween 20 in TBS and once with TBS, and proteins were detected by adding enhanced chemoluminescence solution (Promega, Madison, WI, USA), followed by exposure to medical X-ray film (Agfa Healthcare, Mortsel, Belgium) for 1–10 min.

### Immunoprecipitation

Immunoprecipitation (IP) was performed as previously described [15]. Briefly, we obtained tissue lysates by homogenization in IP buffer (50 mM Tris [pH 7.5], 150 mM NaCl, 5% glycerol, 1% Triton X-100, 1 mM PMSF and 1× protease inhibitor cocktail [Sigma]). The lysates were pre-cleared by incubation in the presence of 20 μL protein A agarose (Pierce™, Thermo Fisher Scientific) at 4 °C for 1 h with constant rotation to remove non-specifically bound proteins. After centrifugation at 20,000 x g for 10 min, the filtered supernatant (500 μg protein) was incubated with 3 μg of the appropriate IP antibody overnight at 4 °C, and then incubated with protein A agarose at 4 °C for 2 h with constant rotation. After washing the beads five times with wash buffer (50 mM Tris [pH 7.5], 150 mM NaCl, 0.2% Triton X-100, 1 mM PMSF, and 1× protease inhibitor cocktail), immunoprecipitated proteins were eluted with buffer containing 2% SDS and subjected to Western blotting.

### Quantative reverse transcriptase-polymerase chain reaction

Under deep inhalation anesthesia, the ipsilateral half of the lumbar (L5–L6) spinal cords of five rats per group were rapidly collected without saline perfusion and immediately frozen in liquid nitrogen. The mRNA levels of IL-1β, IL-6, TNF-α, and IL-2 were measured using quantitative reverse transcriptase real-time PCR (polymerase chain reaction). The tissues were homogenized and total RNA was extracted using TRIzol reagent (T9424; Sigma) according to the manufacturer’s protocol. For quantitative reverse transcriptase real-time PCR (qPCR), 2 μg total RNA were reverse-transcribed by M-MLV reverse transcriptase (Promega); 2 μL RT product was employed as a PCR template. qPCR was performed using iQ SYBR Green SuperMix and the iCycleriQTM Real-Time PCR Detection System (Bio-Rad). PCR conditions consisted of denaturation at 95 °C for 3 min, followed by 40 cycles of denaturation at 95 °C for 15 s, annealing at 58 °C for 15 s, and extension at 72 °C for 30 s. A dissociation curve was generated at the end of each cycle to verify amplification of a single product. mRNA levels were quantified using the 2^-ΔΔC^_T_ method [16]. The mRNA level of the target gene was normalized to that of the housekeeping gene, GAPDH. The gene-specific primers used are listed in Table 1.

**Table 1:** Sequences of the primers used in this study

Primer	Sequence	
*Interleukin-1β* (Gene ID, 24494)	Forward	5′- TGTGATGAAAGACGGCACAC
	Reverse	5′- CTTCTTCTTTGGGTATTGTTTGG
*Interleukin-6* (Gene ID, 24498)	Forward	5′- CCCTTCAGGAACAGCTATGAA
	Reverse	5′- ACAACATCAGTCCCAAGAAGG
*TNF-α* (Gene ID, 24835)	Forward	5′- CCAGGAGAAAGTCAGCCTCCT
	Reverse	5′- TCATACCAGGGCTTGAGCTCA
*Interleukin-2* (Gene ID, 116562)	Forward	5′- AAACTCCCCATGATGCTCAC
	Reverse	5′- GAAAATTTCCAGCGTCTTCCA
*GAPDH* (Gene ID, 24383)	Forward	5′- GAACATCATCCCTGCATCCA
	Reverse	5′- CCAGTGAGCTTCCCGTTCA

### Immunohistochemistry

Immunohistochemical staining was performed as previously described [17]. Rats were deeply anesthetized using 4% isoflurane in 100% oxygen and transcardial perfusion was performed with 250 mL 0.9% normal saline, followed immediately by 4% paraformaldehyde in 0.1 M phosphate-buffered saline (PBS) for 5 min. The harvested L5–L6 spinal cords were post-fixed for 3 h in 4% PFA and immersed in 20% sucrose in 0.1 M PBS overnight. Next, the L5–L6 spinal cords were rapidly frozen in liquid nitrogen and stored at − 80 °C until required for immunohistochemistry. Spinal cords were coronally sectioned on a cryostat to a thickness of 15 μm. The spinal cord sections were rinsed three times with 0.1 M PBS, and non-specific protein binding was blocked by incubation in blocking buffer (2% horse serum, 0.2% Triton X-100, and 0.1% bovine serum albumin in 0.1 M PBS) at 4 °C for 1 h. Next, the sections were incubated overnight at 4 °C with the appropriate primary antibody in 0.1% Triton X-100 in 0.1 M PBS. After washing three times with 0.1 M PBS, the sections were incubated with the corresponding Alexa Fluor-conjugated IgG secondary antibody (Invitrogen, Carlsbad, CA, USA) at a 1:200 dilution for 2 h at room temperature. Negative controls were examined by omitting the corresponding primary antibody. Fluorescence images were acquired using a Zeiss fluorescence microscope (Zeiss, Oberkochen, Germany).

### Statistical analysis

Data are presented as the mean ± SEM, The statistical analysis for the comparisons of changes of the mRNA levels of IL-1β, IL-6, TNF-α, and IL-2 and the results for detecting the development of hyperalgesia of von Frey test were analyzed by Kruskal-Wallis test followed by Tukey’s test using ranks. A *P* value < 0.05 was considered statistically significant.

## Results

### IL-2 expression in the spinal cord ipsilateral to the nerve injury

**Fig. 2:**
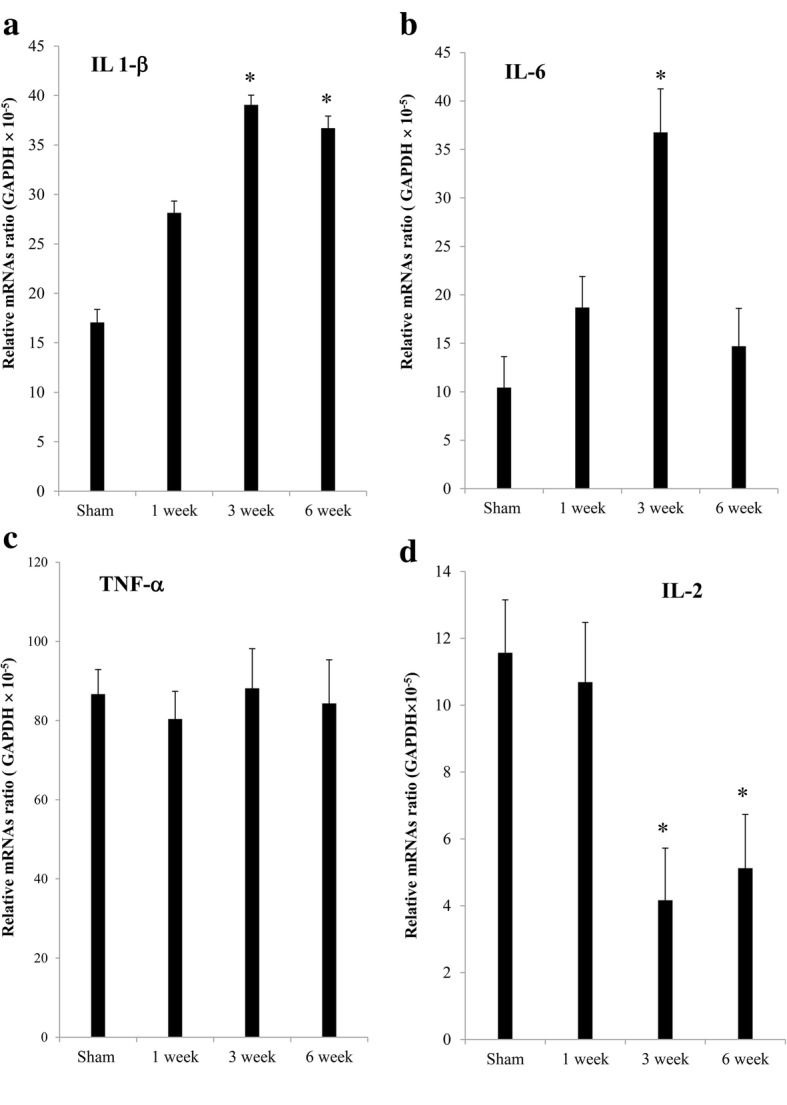
(**a**) IL-1β, (**b**) IL-6, (**c**) TNF-α, and (**d**) IL-2 mRNA levels at 1, 3, and 6 weeks after spinal nerve ligation. IL-2 mRNA levels were significantly decreased at 3 and 6 weeks after spinal nerve ligation. * *P* < 0.05 versus sham-operated group

Pro-inflammatory cytokines such as IL-1β, IL-6, and TNF-α are key factors in the development and maintenance of neuropathic pain and central sensitization [1, 18]. By contrast, IL-2 has antinociceptive effects on neuropathic pain [19]. Therefore, we investigated IL-2, IL-1β, IL-6, and TNF-α mRNA levels in the spinal cord ipsilateral to the nerve injury at 1, 3, and 6 weeks. The mRNA levels of IL-1β and IL-6 were significantly upregulated by 2.3-fold (*P* < 0.05) and 3.5-fold (*P* < 0.05), respectively, compared to those in sham-operated rats at 3 weeks after nerve injury. At 6 weeks, IL-6 but not IL-1β mRNA levels returned to the level of the sham-operated group. TNF-α mRNA levels did not significantly change during the experimental period. These findings are in agreement with the study by Echeverry et al., which used the partial sciatic nerve ligation rodent model [20]. IL-2 mRNA levels at week 1 did not significantly differ from that of sham-operated rats. However, IL-2 mRNA levels were significantly downregulated by 0.36- and 0.44-fold at 3 and 6 weeks, respectively, after nerve injury compared to those of sham-operated rats (Fig. 2).

### Ubiquitin, c-Cbl, and Cbl-b expression in the spinal cord

**Fig. 3:**
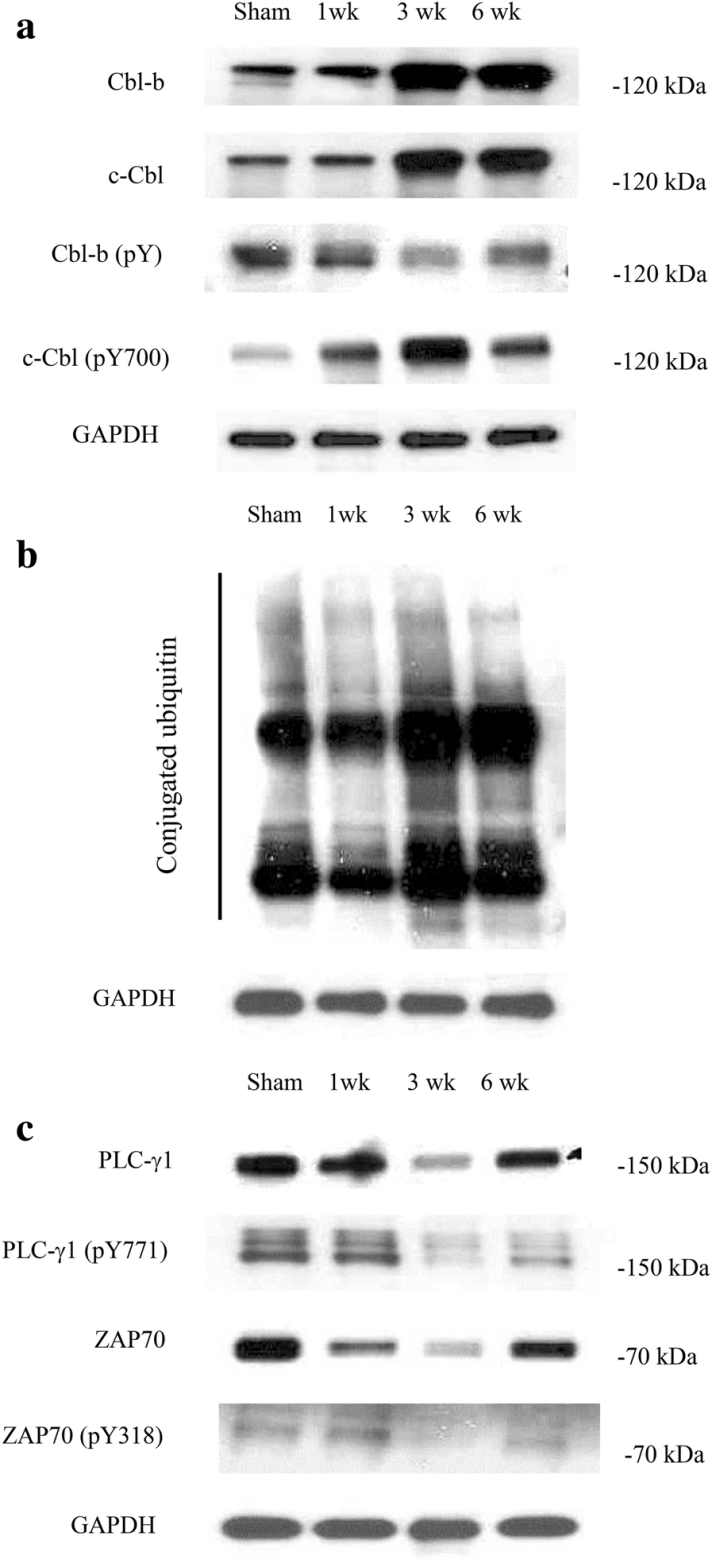
(**a**) Western blot analyses of c-Cbl, Cbl-b, c-Cbl (pY700), and phosphorylated Cbl-b levels at 1, 3, and 6 weeks after spinal nerve ligation. c-Cbl, Cbl-b, and c-Cbl (pY700) levels were significantly increased, and those of phosphorylated Cbl-b were significantly decreased at 3 and 6 weeks after spinal nerve ligation. (**b**) The level of ubiquitin conjugation in the spinal cord ipsilateral to the spinal nerve ligation peaked at 3 weeks. (**c**) Levels of phosphorylated and non-phosphorylated ZAP70 and PLC-γ1 were significantly decreased at 3 and 6 weeks after spinal nerve ligation

Because IL-2 mRNA levels significantly decrease after spinal nerve injury, and IL-2 production is significantly decreased by c-Cbl and Cbl-b in the presence of T cell suppression [10], we investigated Cbl-b and c-Cbl levels after nerve injury by western blotting. At week 1, c-Cbl and Cbl-b levels were not significantly different from those in sham-operated rats, but were significantly upregulated at 3 and 6 weeks after nerve injury (Fig. 3a). At week 1, levels of the phosphorylated forms of Cbl-b and c-Cbl were not significantly different from those of sham-operated rats, but were increased at 3 and 6 weeks. We further investigated whether ubiquitin was increased in parallel, because c-Cbl and Cbl-b are E3 ubiquitin ligases that attach ubiquitin to a target protein. Western blotting showed that the level of ubiquitin conjugation did not increase at week 1, but increased at 3 and 6 weeks (Fig. 3b). These findings suggest that the expression levels of c-Cbl and Cbl-b, as well as ubiquitin conjugation, increased at 3 and 6 weeks after nerve injury.

### Levels of phosphorylated and non-phosphorylated ZAP70 and PLC-γ1

ZAP70 and PLC-γ1 are important members of the RTK signaling pathway involved in IL-2 production, but their interaction with c-Cbl and Cbl-b results in downregulation of the amplitude and duration of RTK signaling [10]. Therefore, we evaluated the levels of phosphorylated and non-phosphorylated ZAP70 and PLC-γ1 by western blotting. At week 1, the levels of phosphorylated and non-phosphorylated ZAP70 and PLCγ1 were not significantly different from those in the sham-operated group, but were decreased at 3 and 6 weeks after nerve injury (Fig. 3c).

### Ubiquitination of PLC-γ1 and protein kinase Cθ

**Fig. 4:**
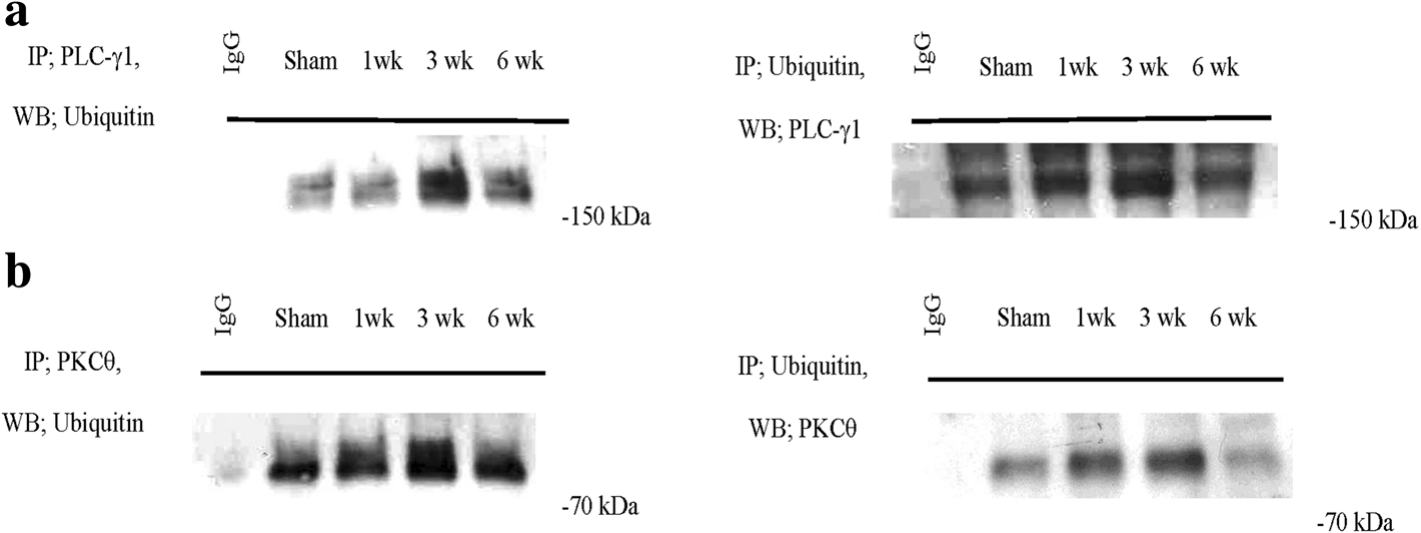
(**a**) PLC-γ1 and (**b**) PKCθ levels were significantly decreased at 3 weeks after nerve injury. The levels of ubiquitination of PLC-γ1 and PKCθ in the spinal cord ipsilateral to the spinal nerve ligation were significantly increased at 3 weeks

PLC-γ1 is a target of Cbl-b and c-Cbl [21]. Increased ubiquitination and proteosomal degradation of PLC-γ1 may lead to decreased IL-2 production. Protein kinase Cθ (PKCθ) and Cbl-b interact bi-directionally (i.e., phosphorylation of Cbl-b by PKCθ leads to its degradation, whereas Cbl-b promotes PKCθ degradation by ubiquitination) [21, 22]. The levels of c-Cbl, Cbl-b, and ubiquitin conjugation were significantly increased, whereas that of phosphorylated PLC-γ1 was decreased at 3 and 6 weeks after nerve injury. Therefore, we evaluated by IP whether ubiquitination of PLC-γ1 was increased after nerve injury. Ubiquitination of PLC-γ1 was not significantly increased at week 1, but its ubiquitination was increased at 3 and 6 weeks after nerve injury (Fig. 4a). This increased PLC-γ1 ubiquitination may be related to the decreased IL-2 production at 3 and 6 weeks after spinal nerve ligation. Moreover, the ubiquitination of PKCθ was increased at 3 and 6 weeks after nerve injury (Fig. 4b), which may enhance the effects of Cbl-b.

### C-Cbl and Cbl-b expression in CD4^+^ T cells

**Fig. 5:**
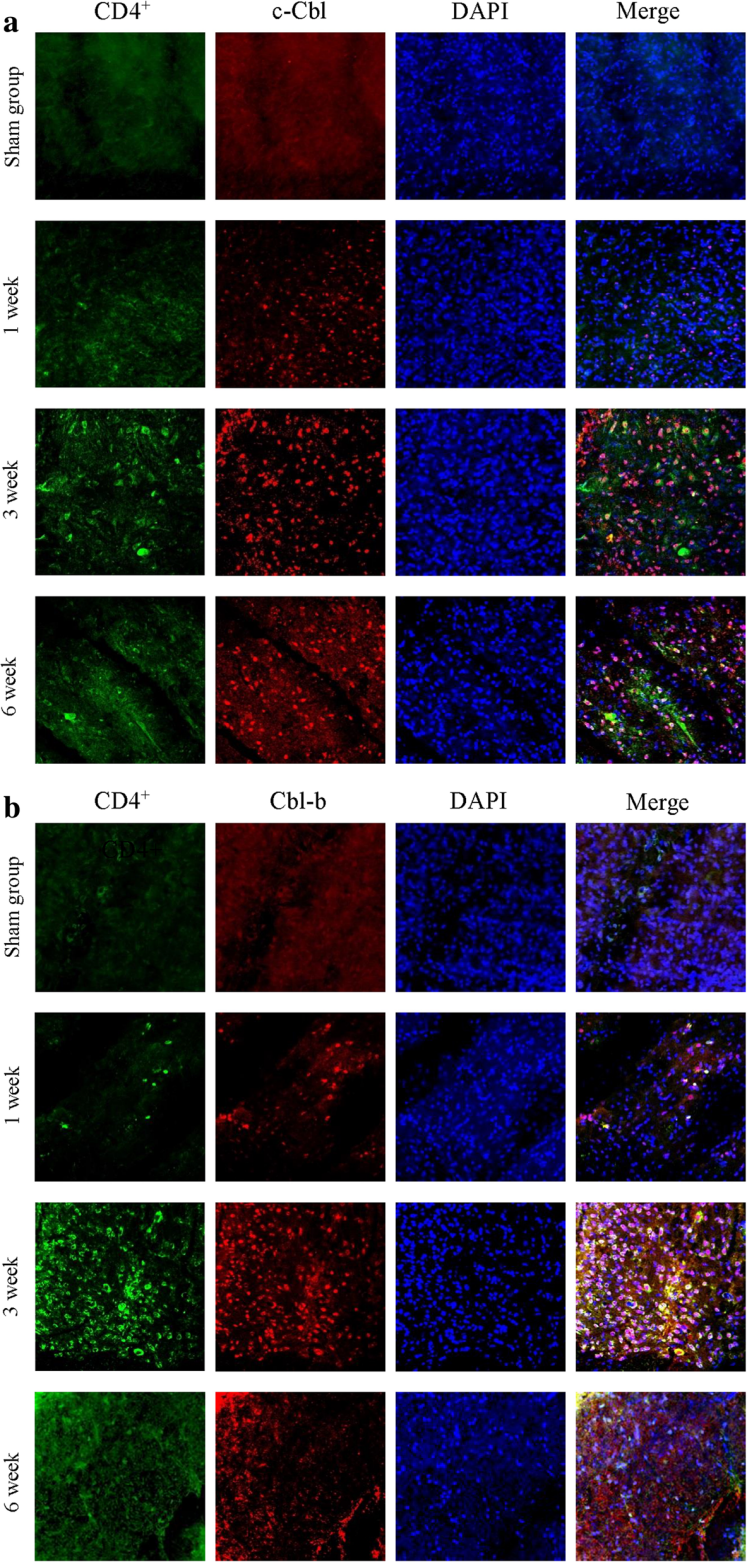
Immunohistochemical analysis of c-Cbl (**a**) and Cbl-b (**b**) expression in CD4^+^ T cells in the ipsilateral dorsal horn area at 1, 3, and 6 weeks after spinal nerve ligation. Infiltration of CD4^+^ T cells into the dorsal horn was slightly increased at 1 week. c-Cbl and Cbl-b levels and the number of CD4^+^ cells infiltrating the ipsilateral dorsal horn area were significantly increased after spinal nerve ligation and peaked at 3 weeks

Although IL-2 expression was decreased and that of c-Cbl and Cbl-b was increased in the ipsilateral spinal cord, the T cell subset affected by the increased expression of c-Cbl and Cbl-b was unclear. Because CD4^+^ T cells produce IL-2 in a manner that is dependent on co-stimulation by CD28^+^ T cells [23], and is influenced by c-Cbl and Cbl-b [24, 25], we investigated c-Cbl and Cbl-b levels in CD4^+^ T cells by immunohistochemistry. CD4^+^ T cell infiltration and c-Cbl and Cbl-b expression in CD4^+^ T cells were not detected in the sham-operated rats. At week 1, CD4^+^ T cell infiltration was detected, and the expression of c-Cbl and Cbl-b was increased, although the differences compared to sham-operated rats were not significant. However, CD4^+^ T cell infiltration was increased in the ipsilateral spinal cord at 3 weeks and decreased at 6 weeks after nerve injury. c-Cbl and Cbl-b levels in CD4^+^ T cells were also significantly increased at 3 weeks, and decreased at 6 weeks, after spinal nerve ligation (Fig. 5).

## Discussion

We demonstrated that IL-2 production is significantly influenced by increased expression of the c-Cbl and Cbl-b E3 ubiquitin ligases in the rat model of neuropathic pain, and the maximum effect occurred at 3 weeks after nerve injury. T cells contribute to the development and maintenance of neuropathic pain symptoms, and pro-inflammatory cytokines secreted by T cells enhance the severity of pain whereas anti-inflammatory cytokines attenuate hypersensitivity to painful stimuli [26–28]. IL-2 is mainly secreted by CD4^+^ T cells, and is well known for its function of maintaining the immune system homeostasis [29]. Austin et al. and Kleinschnitz et al. showed that disturbances of immune system homeostasis aggravate inflammation and pain symptoms following peripheral nerve injury [8, 30] . Our findings showed significantly decreased IL-2 mRNA levels at 3 and 6 weeks after nerve injury. We postulated that decreased production of IL-2 may be associated with aggravated pain symptoms, in addition to its decreased antinociceptive effects, by interfering with the immune system homeostasis.

Ubiquitination, an important post-translational modification by c-Cbl and Cbl-b of ZAP70 and PLC-γ1, results in decreased production of IL-2. E3 ubiquitin ligases transfer ubiquitin to a specific target protein [31, 32], which then is degraded by the ubiquitin-proteasome system and loses its function. In this study, c-Cbl and Cbl-b levels were increased in the subacute phase, but not in the acute phase, of neuropathic pain in a rat model. The delay in the increase in their expression might be related to the delayed infiltration of T cells, which peaked at 3 weeks after nerve injury [7]. Moreover, IL-2 expression decreased in parallel with the increased expression of c-Cbl and Cbl-b at 3 and 6 weeks. Therefore, the increased expression of c-Cbl and Cbl-b in CD4^+^ T cells may reduce production of IL-2 by increasing ubiquitination of ZAP70 and PLC-γ1 in the rat model of neuropathic pain. c-Cbl and Cbl-b belong to the Cbl family and share highly conserved N-terminal regions. c-Cbl and Cbl-b act as linker proteins, but c-Cbl is activated by phosphorylation by Src family protein tyrosine kinases [33], while phosphorylation by Cbl-b results in proteosomal degradation [21]. In this study, phosphorylation of Cbl-b decreased, and ubiquitination of PKCθ increased, at 3 and 6 weeks after nerve injury. When phosphorylation was prevented by increased ubiquitination of PKCθ at 3 and 6 weeks after nerve injury, stronger Cbl-b function and protein stability were observed.

IL-2 has antinociceptive effects by interacting with the μ-opioid receptor [34]. Our results showed that significantly decreased IL-2 levels at 3 and 6 weeks after nerve injury resulted in an increase in the severity of neuropathic pain. IL-2 has been used therapeutically, but is associated with severe side effects [35]. Our findings suggest that regulation of c-Cbl and Cbl-b has the potential for management of neuropathic pain.

## Conclusion

Our results suggest that ubiquitin and the c-Cbl and Cbl-b E3 ubiquitin ligases regulate the development of neuropathic pain by attenuating the production of IL-2. Our findings showed that the pathogenesis of neuropathic pain caused by an imbalance of the cell-mediated immune system peaked in the subacute phase. Therefore, efforts to prevent the development of neuropathic pain after nerve injury should take into consideration the changes in the neuroimmune environment that occur during the subacute phase.

## Abbreviations

PLC-γ1: phospholipase C-γ1
PKCθ: protein kinase Cθ
Cbl: casitas B lineage lymphoma
IL-2: Interleukin 2
IP: immunoprecipitation
PAGE: polyacrylamide gel electrophoresis
PBS: phosphate-buffered saline
PCR: polymerase chain reaction
PVDF: polyvinylidene difluoride
qPCR: quantitative reverse transcriptase real-time PCR
RTK: regulating receptor tyrosine kinase
SDS: sodium dodecyl sulfate
TBS: Tris-buffered saline
Th17: T helper 17
Treg: regulatory T cell
